# Interaction of protocadherin-15 with the scaffold protein whirlin supports its anchoring of hair-bundle lateral links in cochlear hair cells

**DOI:** 10.1038/s41598-020-73158-1

**Published:** 2020-10-02

**Authors:** Vincent Michel, Elise Pepermans, Jacques Boutet de Monvel, Patrick England, Sylvie Nouaille, Alain Aghaie, Florent Delhommel, Nicolas Wolff, Isabelle Perfettini, Jean-Pierre Hardelin, Christine Petit, Amel Bahloul

**Affiliations:** 1 Institut Pasteur, Unité de génétique et physiologie de l’audition, 75015 Paris, France; 2 UMRS 1120, Institut National de la Santé et de la Recherche Médicale, 75015 Paris, France; 3 Complexité du vivant, Sorbonne Université, 75005 Paris, France; 4 Institut de l’audition, Centre de l’Institut Pasteur, 75012 Paris, France; 5 Institut Pasteur, Plateforme de biophysique moléculaire, Paris, France; 6 Institut de la vision, Syndrome de Usher et autres atteintes rétino-cochléaires, 75012 Paris, France; 7 Institut Pasteur, Unité Récepteurs-Canaux, 75015 Paris, France; 8 Collège de France, 75005 Paris, France; 9 Present Address: Center for Proteomics, University of Antwerp, 2020 Antwerp, Belgium; 10 Present Address: Institute of Structural Biology, Helmholtz Zentrum, Munich, Germany; 11 Present Address: Department of Otolaryngology, Head and Neck Surgery, Stanford University, Stanford, CA USA

**Keywords:** Biochemistry, Neuroscience, Diseases

## Abstract

The hair bundle of cochlear hair cells is the site of auditory mechanoelectrical transduction. It is formed by three rows of stiff microvilli-like protrusions of graduated heights, the short, middle-sized, and tall stereocilia. In developing and mature sensory hair cells, stereocilia are connected to each other by various types of fibrous links. Two unconventional cadherins, protocadherin-15 (PCDH15) and cadherin-23 (CDH23), form the tip-links, whose tension gates the hair cell mechanoelectrical transduction channels. These proteins also form transient lateral links connecting neighboring stereocilia during hair bundle morphogenesis. The proteins involved in anchoring these diverse links to the stereocilia dense actin cytoskeleton remain largely unknown. We show that the long isoform of whirlin (L-whirlin), a PDZ domain-containing submembrane scaffold protein, is present at the tips of the tall stereocilia in mature hair cells, together with PCDH15 isoforms CD1 and CD2; L-whirlin localization to the ankle-link region in developing hair bundles moreover depends on the presence of PCDH15-CD1 also localizing there. We further demonstrate that L-whirlin binds to PCDH15 and CDH23 with moderate-to-high affinities in vitro*.* From these results, we suggest that L-whirlin is part of the molecular complexes bridging PCDH15-, and possibly CDH23-containing lateral links to the cytoskeleton in immature and mature stereocilia.

## Introduction

The sensory cells of the cochlea (inner and outer hair cells) convert acoustic waves into receptor potentials by the process of mechanoelectrical transduction (MET)^1,2^. This process takes place in the hair bundle, a mechanosensitive antenna formed by thick and stiff microvilli-like protrusions called stereocilia, organized in three rows of graduated height (i.e., short, middle-sized, and tall stereocilia) at the apical surface of the hair cells. Stereocilia are connected, within and between rows, by various types of fibrous links, expressed both in the developing and the mature cochlea. According to the current view of the MET process, cationic transducer channels located at the tips of short and middle-sized stereocilia^3^ are gated by the sound-evoked periodic tension of the tip link, an oblique link connecting the tip of each short and middle-sized stereocilium to the shaft of the adjacent taller stereocilium, to which these channels are mechanically coupled^2,4,5^. In addition, horizontal top connectors in outer hair cells (OHCs) or loosely defined lateral links in inner hair cells (IHCs) connect all stereocilia in mature hair cells^6–8^. In developing hair bundles, the transient lateral links consist of multiple connectors, located along the stereocilium shafts from the earliest stages of hair bundle growth (starting around embryonic day (E) 16 in mice), and the ankle links, located near the stereocilia base from around postnatal day (P) 2 to P9 in mice^9^. In addition, some lateral links connect the tallest stereocilia to the transient kinocilium^10^. Several lines of evidence indicate that the transient lateral links are involved in the cohesiveness of growing hair bundles^11^, and that the ankle-links are involved in the final shaping of hair bundles before the onset of hearing (which occurs on P12-P13 in mice)^9^.

Certain proteins forming or associated with the above-mentioned interstereociliary links have been identified. Two cadherins, protocadherin15 (PCDH15) and cadherin23 (CDH23), form the lower and upper parts of the tip link, respectively^12^. These proteins also form transient lateral links in the developing hair bundle^13–15^. Likewise, current evidence indicates that usherin and adhesion G protein-coupled receptor V1 (ADGRV1), two large transmembrane proteins, are components of the ankle links^9^, while stereocilin, otogelin and otogelin-like are associated with the OHC horizontal top connectors^16,17^, and protein tyrosine phosphatase receptor type Q (PTPRQ) is associated with shaft connectors^18^. By contrast, the proteins bridging the various types of links to the dense F-actin cytoskeleton of the stereocilia remain largely unknown, despite a number of putative candidates. Indeed, the stability of these links likely involves protein complexes located between the plasma membrane and actin filaments. Current evidence indicates that the submembrane scaffold proteins harmonin (a PDZ-domain containing protein) and SANS (a protein containing ankyrin repeats), together with the motor protein myosin VIIa, are responsible for the anchoring of the upper part of the tip link (formed by CDH23) to the actin filaments^13,19–21^. While a similar connection may exist between the lower part of the tip link (formed by PCDH15) and F-actin, its molecular composition is still elusive. In the developing hair bundles, two other PDZ-domain containing scaffold proteins, whirlin and PDZD7, are associated with the ankle links, and have been shown to bind to the cytoplasmic domains of usherin and ADGRV1 in vitro^9,21,22^.

**Figure 1:**
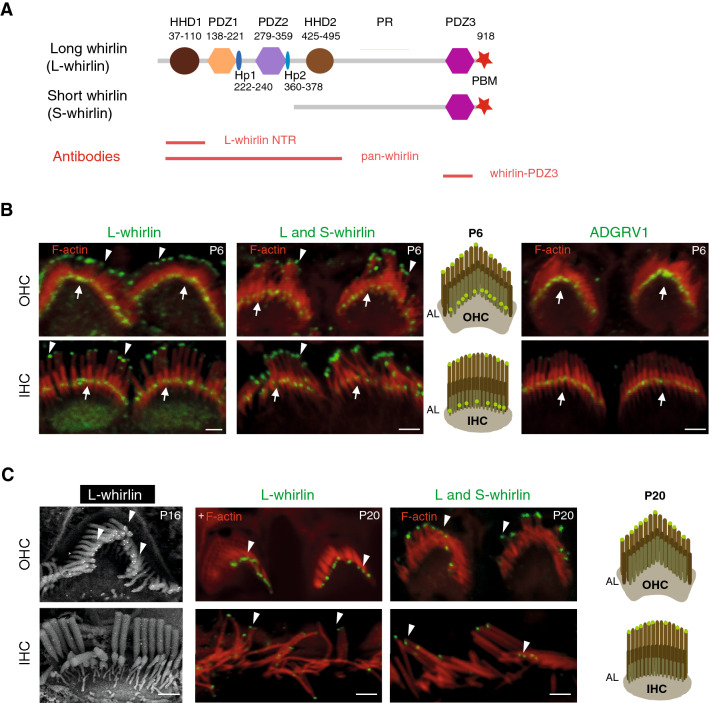
Distribution of L-whirlin in the developing and mature hair bundles of the mouse cochlea. (**A**) Schematic representation of the L-whirlin and S-whirlin isoforms. Red lines indicate the positions of the segments used to produce antibodies against L-whirlin and against both whirlin isoforms. (**B**) Whirlin immunolocalization in developing hair bundles. Left and middle panels: confocal images of OHC and IHC hair bundles stained for either L-whirlin (anti-L-whirlin NTR antibody; left panel, green) or both whirlin isoforms (anti-whirlin-PDZ3 antibody; middle panel, green) and for actin (red) on P6. The immunoreactivity is detected at the same places with the two antibodies, namely at the tips of OHC and IHC tall stereocilia (arrowheads), and in the ankle-link region (AL) of all stereocilia (white arrows). Right panel: confocal images of OHCs and IHCs immunolabeled for the ankle-link protein ADGRV1 (green; white arrows) on P6. Scale bar: 2 µm. (**C**) Whirlin immunolocalization in mature hair bundles. Left panel: Scanning electron micrograph of OHC and IHC hair bundles immunostained for L-whirlin on P16. Protein A conjugated to 15 nm-diameter gold particles was used for detection. The labeling is detected only at the tips of OHC tall stereocilia (arrowheads). Middle and right panels: confocal microscopy immunolabeling of L-whirlin (middle panel) and of both whirlin isoforms (right panel) on P20. The labeling is present at the tips of OHC and IHC tall stereocilia (arrowheads) with the two antibodies. Scale bar: 2 µm.

Cochlear hair cells produce at least two different isoforms of whirlin^23–25^. The so-called long isoform, hereafter referred to as L-whirlin, is closely related to harmonin^23^. It consists of an N-terminal region (NTR) including a harmonin-homology domain (HHD1), two PDZ domains (PDZ1, PDZ2), a second harmonin-homology domain (HHD2), a proline-rich domain (PR), a third PDZ domain (PDZ3), and a C-terminal PDZ domain-binding motif (PBM). The so-called short isoform, referred to as S-whirlin, has the same C-terminal region as L-whirlin, but does not extend beyond the PR domain on its N-terminal side (Fig. 1A). Mutant mice lacking both L- and S-whirlin isoforms (*Whrn*^wi/wi^ mice, known as whirler mice) are profoundly deaf due to abnormally short stereocilia and misshaped hair bundles in IHCs and OHCs^26^. Attempts to rescue these mice from their abnormal hair bundle phenotype with a transgene producing the S-whirlin isoform were successful in correcting the stereocilia size defects and misshaping of IHC hair bundles, but not of OHC hair bundles^23^. Accordingly, in mutant mice lacking only the L-whirlin isoform (*Whrn*^neo/neo^ mice), OHC hair bundles have an abnormally round (‘U’-like) shape^24^, and distortion product otoacoustic emissions (DPOAEs) and frequency tuning are affected, indicating impaired OHC function in these mice^25^. In addition, the stereocilia of mature IHCs appear abnormally thick^24^. There is indirect evidence that at least S-whirlin is involved in the polymerization and stabilization of actin filaments at the tips of the tall stereocilia^23,27–31^. However, the distribution and role of L-whirlin in the hair bundles of mature hair cells are still unclear. To address these issues, we reanalyzed the location of L-whirlin in the cochlear sensory epithelium (organ of Corti) by immunofluorescence, and characterized its interaction with PCDH15 isoforms and CDH23 in surface plasmon resonance assays.

## Results

### L-whirlin localization in the developing hair bundle critically depends on the presence of PCDH15 and CDH23

**Figure 2:**
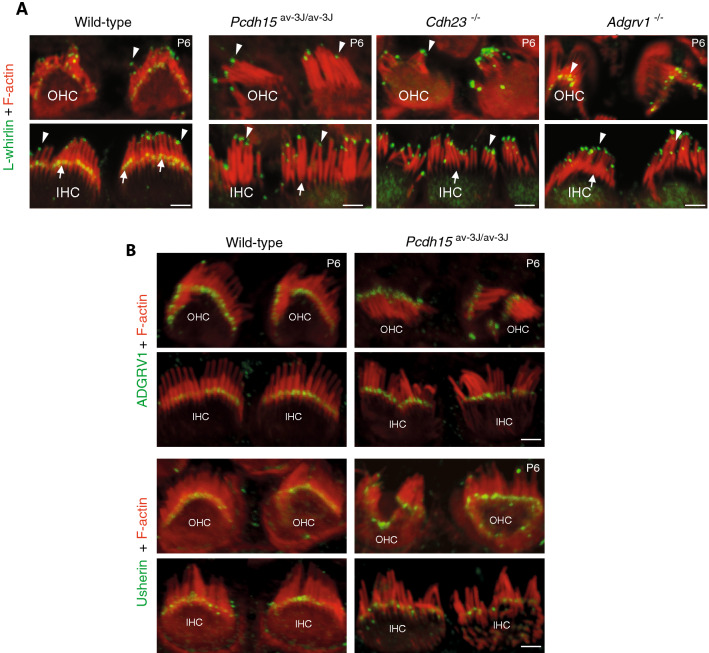
Localization of L-whirlin in the cochlear hair bundles of wild-type mice and in *Pcdh15*^av-3J/av-3J^, *Cdh23*^−/−^, and *Adgrv1*^−/−^ mutant mice. (**A**) The L-whirlin immunolabeling of the ankle-link region of stereocilia in the wild-type mouse is not detected in the *Pcdh15*^av-3J/av-3J^, *Cdh23*^−/−^, and *Adgrv1*^−/−^ mice on P6 (white arrows), whereas the apical labeling persists in the mutant mice (arrowheads). Scale bar: 2 µm. (**B**) Confocal images of hair cells immunostained for either ADGRV1 (above) or usherin (below) (green), and stained for actin (red), in wild-type and *Pcdh15*^av-3J/av-3J^ P6 mice. The ADGRV1 and usherin labelings of the ankle-links region are detected both in the wild-type mouse and in the *Pcdh15*^av-3J/av-3J^ mutant mouse. Scale bar: 2 µm.

A variety of *Whrn* transcripts, including transcripts encoding the predicted L-whirlin and S-whirlin isoforms, have been reported in the mouse inner ear^23–25,32^. There is consistent immunolabeling evidence of the presence of both isoforms at all stages in the IHCs^24,25^ and at developmental stages in the OHCs; some evidence of whirlin labeling also exists in mature OHCs (^33^ and Table S1). The localization of L-whirlin has remained, however, controversial. Using an antibody specific for this isoform, one group observed its presence at the base of stereocilia in the developing hair bundles of IHCs and OHCs, and at stereocilia tips in developing and mature IHCs only^24^. Another group, combining observations made by structured illumination microscopy in wild-type mice and in L-whirlin-null mutant mice with several antibodies recognizing both the L- and S-isoforms, concluded that L-whirlin is restricted to a position midway along the middle-sized stereocilia of both IHCs and OHCs on P10^25^. It is unclear whether this discordance was due to the different epitopes recognized by the antibodies used, or to differences between the developmental stages analyzed. We addressed this issue by producing a new antibody directed specifically against the N-terminal region of L-whirlin (L-whirlin NTR; see Materials and methods). The specificity of this antibody was validated by western blot experiments with protein extracts from transfected cells, and by the absence of immunolabeling of the cochlear hair cells in whirler (*Whrn*^wi/wi^) mutant mice lacking all whirlin isoforms (Fig. S1). We also produced two antibodies recognizing both whirlin isoforms (pan-whirlin and whirlin-PDZ3; Fig. 1 and Materials and methods). The labeling obtained in the organ of Corti with these antibodies is consistent with previously reported data using other non-isoform specific anti-whirlin antibodies^24,25^ (Fig. S1). In P6-P7 wild-type mice, using the L-whirlin NTR antibody, we observed a labeling of the hair bundle bases in IHCs and OHCs, and of the tips of the tall stereocilia in IHCs, consistent with the findings of Mathur and colleagues^24^. However, we also found a labeling of the tips of the tall stereocilia in OHCs (Fig. 1B; Fig. S1A). The L-whirlin immunoreactivity of the tips of the tall stereocilia was still present in both IHCs and OHCs on P20, and was observed by using immunogold scanning electron microscopy of OHC hair bundles on P16 (Fig. 1C). The immunoreactivity seen earlier at the base of IHC and OHC stereocilia was no longer present on P16, which is consistent with the previously reported contribution of L-whirlin to the transient ankle-link complex^24^ (Fig. 1C). Accordingly, we could not detect any L-whirlin labeling in the stereocilia ankle-link region of P6 *Adgrv1*^−/−^ mutant mice that lack ADGRV1 and the ankle links, whereas the protein was still detected at the tips of the tallest stereocilia of both IHCs and OHCs in these mice (^9^ and Fig. 2A). Because PCDH15 and CDH23 form transient lateral links, and have been detected on P5 in the stereocilia basal region and at the tips of the tall stereocilia, respectively (using, for PCDH15, antibodies specific for the CD1 isoform)^15,34^, we analyzed the distribution of L-whirlin in *Pcdh15*^av-3J/av-3J^ and in *Cdh23*^−/−^ mutant mice, which lack all transmembrane PCDH15 and CDH23 isoforms, respectively^35,36^. In these two mutant mice, L-whirlin immunoreactivity of the ankle link region of IHC and OHC stereocilia was undetectable on P6, whereas immunoreactivity of the tips of the tallest stereocilia was unaffected (Fig. 2A). Notably, the immunoreactivity of the ankle link region for usherin and ADGRV1 was little affected in *Pcdh15*^av-3J/av-3J^ mutant mice, forming a neat line at the stereocilia base (Fig. 2B), which is consistent with the previously reported preservation of the ankle links in these mice^34^. These results suggest that the localization of L-whirlin at the ankle links, but not at the tips of the tall stereocilia, is critically dependent on the presence of PCDH15 in this region, and possibly also of CDH23. However, the severe developmental abnormalities of the hair bundles in the absence of CDH23 could clearly affect the integrity of the ankle links, which could also prevent the localization of L-whirlin in this region.

### Distribution of the different PCDH15 isoforms in the hair bundles of cochlear hair cells

**Figure 3:**
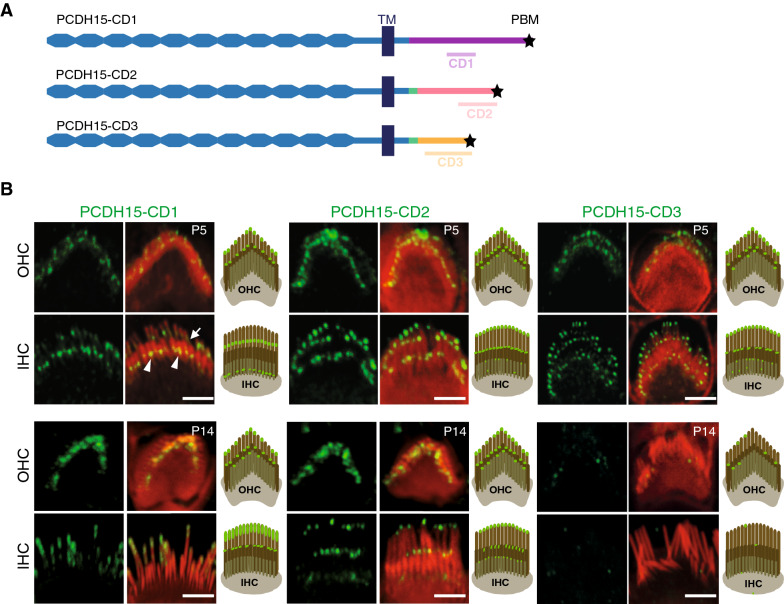
Distribution of the different PCDH15 isoforms in developing and mature hair bundles of the mouse cochlea. (**A**) Diagram of transmembrane PCDH15 isoforms. The positions of the protein fragments used to produce the anti-PCDH15 antibodies are indicated (TM: transmembrane domain, * PBM: PDZ domain-binding motif). The short cytoplasmic fragment common to all PCDH15 isoforms is drawn in blue, and the following shorter fragment (encoded by exon 37) common to CD2 and CD3 isoforms is drawn in green. Finally, the C-terminal fragments specific to CD1, CD2, and CD3 isoforms are drawn in purple, pink, and yellow, respectively. (**B**) Confocal microscopy images of the organ of Corti (whole-mounts) and diagrams of hair bundles (OHC above, IHC below) immunostained for PCDH15- CD1, CD2, or CD3 isoforms (green) and stained for actin (red) on P5 and P14. In the top left panel, arrowheads and arrows indicate the PCDH15-CD1 immunostaining of the ankle link region and of a subapical region of the tall stereocilia, respectively. Scale bar: 2 µm.

Until now, the immunolocalization results reported in immature cochlear hair cells for the three different groups of transmembrane PCDH15 isoforms (CD1, CD2, and CD3) have been inconclusive^34,37,38^. We analyzed the distribution of these isoforms in the hair bundles of P5-P7 murine IHCs and OHCs with antibodies specific to each isoform group (Fig. 3A; Figs. S2, S3, S4; see Materials and methods). In the P5 and P7 OHCs, CD1, CD2, and CD3 immunolabelings were restricted to the apices of all stereocilia, which is consistent with the location of the tip-link lower insertion point on the short and middle-sized stereocilia, and of additional apical links in the three rows of stereocilia (Fig. 3B and Fig. S5). On P5, CD2 and CD3 labelings in the IHCs were the same as in OHCs (Fig. 3B). In accordance to the previously reported immunoreactivity of the ankle link region for PCDH15-CD1^34^, an immunolabeling at the base of IHC stereocilia at P5 and P7 was detected for the PCDH15-CD1 isoform. An additional labeling was observed in the subapical region of the tall stereocilia (Fig. 3B and Fig. S5A). On P9, the CD3 staining had disappeared in the IHCs and OHCs of the basal region of the cochlea (Fig S5B), where hair cell maturation proceeds first^39^. In mature hair cells, PCDH15-CD2 is known to form the lower part of the tip links^40^, but the localizations of the CD1 and CD3 isoforms have remained unresolved^37^. On P14, the CD2 isoform was detected at the tips of all stereocilia, including the short and middle-sized stereocilia and the tall stereocilia, both in IHCs and OHCs (Fig. 3B and^40^). The immunolocalization of the CD1 isoform was also restricted to the apical region of all stereocilia (including the tall ones), but the labeling of the tall IHC stereocilia extended to more distant locations below the tip than it did for the CD2 isoform (Fig. 3B). In contrast with the observations of Ahmed and collaborators, the murine cochlea was no longer immunoreactive for PCDH15-CD3 on P14 (Fig. 3B), which could be caused by a masked and inaccessible epitope, or interspecies differences^37^. From these results, we infer that the CD1 and CD2 isoforms of PCDH15 could also form lateral links in the apical parts of mature stereocilia of the three rows, both in IHCs and OHCs.

### L-whirlin is a binding partner of PCDH15 and CDH23

**Figure 4:**
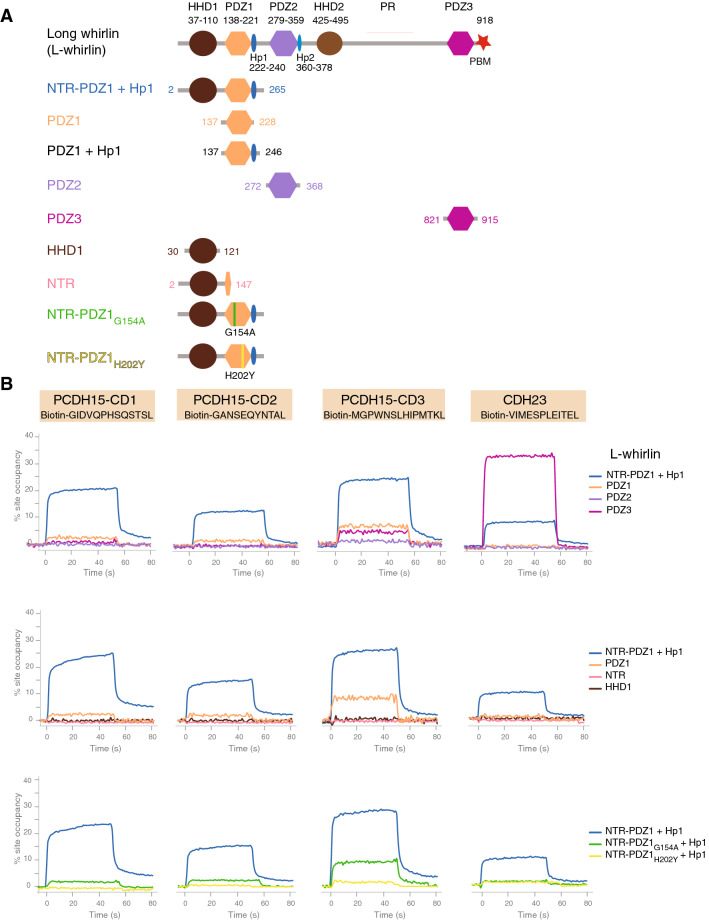
L-whirlin interacts with the three PCDH15 isoforms in vitro. (**A**) Overview of the different whirlin protein constructs used in the binding experiments (whirlin-NTR-PDZ1-Hp1: aa residues 2–265, whirlin-PDZ1: aa residues 137–228, whirlin PDZ1-Hp: aa residues 137-246, whirlin-PDZ2: aa residues 272–368, whirlin-PDZ3: aa residues 821–915, whirlin-HHD1: aa residues 30–121, whirlin-NTR: aa residues 2–147; Genbank accession number Q80VW5). (**B**) SPR sensograms showing responses curves (% site occupancy as a function of time) to different whirlin protein constructs at 10 μM on different peptides (immobilized via their N-terminal Biotin tag) matching the C-terminal sequences of PCDH15-CD1, PCDH15-CD2, PCDH15-CD3, and CDH23 (PCDH15-CD1: biotin-TTDS-GIDVQPHSQSTSL; PCDH15-CD2: biotin-TTDS-GANSEQYNTAL; PCDH15-CD3: biotin-TTDS-MGPWNSLHIPMTKL; CDH23: biotin-TTDS-VIMESPLEITEL). The PCDH15 C-terminal peptides show the highest interaction signal with the NTR-PDZ1-Hp1 fragment of whirlin, whereas the CDH23 C-terminal peptide shows the highest interaction signal with the whirlin PDZ3 domain (top panels). The NTR and HHD1 whirlin fragments do not interact with the PCDH15 or CDH23 C-terminal peptides (middle panels). Substitution of the highly conserved GLGF motif by GLAF in the NTR-PDZ1_G154A_ mutant markedly reduces interactions with the PCDH15 and CDH23 C-terminal peptides, and changing the predicted type I PDZ1 domain of whirlin to a type II PDZ domain in the NTR-PDZ1_H202Y_ mutant completely suppressed these interactions (bottom panels). These mutations did not alter the protein folding analyzed by circular dichroism (see Fig. S9).

Based on the above-mentioned loss of the ankle-link complex immunoreactivity for L-whirlin in mutant mice lacking PCDH15, and the simultaneous presence of at least two different PCDH15 isoforms and L-whirlin at the tips of the tall stereocilia in mature cochlear hair cells, we first investigated a possible interaction of L-whirlin and S-whirlin with the various transmembrane isoforms of PCDH15 in vitro. These isoforms are classified into three classes (CD1, CD2, CD3) differing by their cytoplasmic domains, which all show an isoform-specific C-terminal PBM. We measured the binding affinities between synthetic C-terminal peptides (including the PBM), which mimic the C-terminal sequences of the three transmembrane PCDH15 isoforms (13 aa for CD1; 11 aa for CD2; 14 aa for CD3), and protein fragments containing the different PDZ domains of whirlin by the surface plasmon resonance technique. Because it has been suggested that the NTR-PDZ1 domain of L-whirlin form a functional supramodule, and that the Hp1 domain (an 18 amino acid hairpin extension of the PDZ1 domain) contributes to stabilizing the binding of the PDZ1 domain to its ligand^41^, we used two additional constructs, the first one consisting of the NTR-PDZ1 domain, and the second one of a protein fragment containing the NTR, PDZ1 and Hp1 domains (Fig. 4; Table 1; Figs. S6, S7, S8). We found that the three PCDH15 peptides (referred to as PCDH15-CD1, PCDH15-CD2, and PCDH15-CD3) had similar moderate-to-high affinity binding to this NTR-PDZ1-Hp1 whirlin fragment (dissociation constants, K_d_, values of 8.8 μM for PCDH15-CD1, 8.5 μM for PCDH15-CD2, and 4.2 μM for PCDH15-CD3), but only low affinity interactions with PDZ2 and PDZ3 (K_d_ > 200 μM). From these results, we conclude that the different PCDH15 isoforms may interact specifically with the NTR-PDZ1-Hp1 fragment of L-whirlin also in vivo.

**Table 1:** Dissociation constants (*K*_d_) of the interactions between different whirlin constructs and the PCDH15 and CDH23 C-terminal peptides.

Whirlin fragments	PCDH15-CD1	PCDH15-CD2	PCDH15-CD3	CDH23
NTR-PDZ1 + Hp1	8.8 ± 0.2 μM	8.5 ± 1 μM	4.2 ± 0.1 μM	11.3 ± 0.8 μM
PDZ1 + Hp1	29 ± 3 μM	33 ± 2 μM	11 ± 1 μM	64 ± 6 μM
PDZ1	106 ± 25 μM	147 ± 50 μM	34 ± 3 μM	141 ± 80 μM
PDZ2	> 1 mM	> 1 mM	460 ± 50 μM	> 1 mM
PDZ3	> 1 mM	> 1 mM	230 ± 30 μM	4.3 ± 0.3 μM
HHD1	> 1 mM	> 1 mM	> 1 mM	> 1 mM
NTR	> 1 mM	> 1 mM	> 1 mM	> 1 mM

Different concentrations of the whirlin fragments were injected as analytes in a randomized order on the different peptides (immobilized as ligands). *K*_d_ values were determined for each analyte-ligand interaction. They are indicated in the table as mean ± s.d. of three different experiments. The raw data corresponding to the interaction of the different whirlin constructs with PCDH15 and CDH23 C-terminal peptides are shown in Figs. S6, S7 and S8.

We proceeded to an analysis of the contributions of the three domains of the L-whirlin NTR-PDZ1-Hp1 fragment to this interaction. We did not detect any interaction between any of the three PCDH15 peptides and either the entire NTR of whirlin (containing the HHD1 domain) or the HHD1 domain alone (K_d_ > 1 mM) (Fig. 4; Table 1; Figs. S7, S8). The whirlin PDZ1 domain alone did bind to CD1, CD2, and CD3, but with much lower affinities (K_d_ values of 106 μM for PCDH15-CD1, 147 μM for PCDH15-CD2, and 34 μM for PCDH15-CD3) than those measured with the entire NTR-PDZ1-Hp1 fragment. Finally, we tested the whirlin PDZ1-Hp1 fragment, and found higher binding affinities (K_d_ values of 29 μM for PCDH15-CD1, 33 μM for PCDH15-CD2, and 11 μM for PCDH15-CD3) than with the PDZ1 domain alone, but still lower than those measured with the entire NTR-PDZ1-Hp1 fragment (Table 1). These results support the notion that the NTR-PDZ1-Hp1 fragment of whirlin forms a functional supramodule similar to that of harmonin^20,42^. The binding of the PCDH15 C-terminal peptides to the NTR-PDZ1-Hp1 fragment of whirlin was drastically reduced or completely abolished upon substitution, in the PDZ1 domain, of two different residues at amino acid positions critically involved in canonical PDZ-PBM interactions^43^ (Fig. 4; Figs. S7, S8). These substitutions did not affect, however, the folding of the PDZ1 domain as analyzed by circular dichroism (Fig. S9). We therefore conclude that (1) the binding of the PCDH15 C-terminal peptides to the NTR-PDZ1-Hp1 fragment of whirlin is consistent with a canonical PDZ-PBM interaction, and (2) the whirlin NTR domain (containing the HHD1 domain) and the PDZ1 domain with its hairpin extension (Hp1) both contribute to the stabilization of this interaction, presumably by facilitating an efficient exposure of the PDZ1 domain critical residues to the PBM of the PCDH15 isoforms.

The PDZ2 domain of harmonin has been found to interact in pull-down assays with protein fragments encompassing the entire cytoplasmic region of PCDH15-CD1, but the affinity of this interaction has not been quantified^34,44,45^. We used the surface plasmon resonance technique to determine if the different PCDH15 C-terminal peptides (13 aa for CD1; 11 aa for CD2; 14 aa for CD3) also bind to harmonin fragments in vitro. By this technique, only weak interactions were detected between the C-terminal peptides of the three PCDH15 isoforms and any of the PDZ domains of harmonin (individually, or in tandem association) or the full-length harmonin-a1 protein, all K_d_ values being above 200 μM (Fig. S10 and data not shown) although high affinity interactions were detected between the NTD-PDZ1-Hp and PDZ2 domains of harmonin and the C-terminal residues of SANS (ALERPLALEDTEL) and CDH23 (MGPWNSLHIPMTKL) respectively. This indicates that PCDH15 is unlikely to bind to harmonin through a conventional PDZ-PBM interaction in vitro or in vivo.

We also investigated a possible interaction of L-whirlin and S-whirlin with CDH23 in vitro.

We measured the binding affinities between a synthetic peptide matching the C-terminal residues of CDH23, including the PBM, and different whirlin fragments (NTR-PDZ1-Hp1, PDZ2 domain, and PDZ3). We found moderate-to-high binding affinities with the Nter-PDZ1-Hp1 fragment, specific to L-whirlin (K_d_ ~ 20 μM), and with the PDZ3 domain, common to L- and S-whirlin (K_d_ ~ 4 μM) (Fig. 4; Table 1; Figs. S6, S8), but did not detect any interaction with the PDZ2 domain. From these results, we conclude that CDH23, unlike PCDH15, could interact with both L- and S-whirlin in vivo.

## Discussion

We show here that L-whirlin binds to the C-terminal parts of all three PCDH15 isoforms in vitro, through a canonical PDZ-PBM interaction involving the first PDZ domain (PDZ1) of L-whirlin. The measured affinities were moderate-to-high (in the 1 to 10 µM range), making PCDH15 isoforms plausible binding partners of L-whirlin in cochlear hair cells.

The mild disorganization of the hair bundles in mutant mice defective for L-whirlin suggests that L-whirlin is not critical in the early formation of the hair bundle, but is involved in its late shaping. We cannot exclude the possibility that another scaffold protein binding to PCDH15 compensates for the absence of L-whirlin in these mutants. However, the involvement of harmonin in such a compensation process via PDZ/PBM interaction appears unlikely due to the low binding affinities (K_d_ values above 200 µM) that we found between the PCDH15 C-terminal peptides (including the PBMs) and any of the three PDZ domains of harmonin, or even the full-length harmonin-a, by the surface plasmon resonance technique, even though the harmonin PDZ2 domain had previously been reported to interact with the cytoplasmic region of PCDH15-CD1 in pull-down assays^44,45^. We also show that the CDH23 PBM binds to the PDZ1 domain of L-whirlin and to the PDZ3 domain common to both whirlin isoforms with moderate-to-high affinities in vitro, in addition to its previously reported interaction with harmonin^20,42^.

By using a new antibody specific to L-whirlin, we confirmed that during hair bundle development, L-whirlin localizes to the ankle link region, and could therefore be associated with these links. In addition, L-whirlin was not detected in the ankle link region of mutant mice lacking PCDH15 or CDH23, which indicates that these two unconventional cadherins play important and previously unsuspected roles in the ankle-link complex during hair bundle development. Considering the preserved status of the ankle links in *Pcdh15*^av-3J/av-3J^ mice^34^, confirmed by the persistence of the immunoreactivity for ADGRV1, an ankle link component, in these mice (Fig. 2B), we suggest that the localization of L-whirlin in the ankle link region involves the presence of PCDH15, and thus possibly of PCDH15-containing lateral links, also at this location. Unlike for PCDH15, evidence for the presence of CDH23 in the ankle link region is lacking, and the reason for the disappearance of L-whirlin from this region in CDH23-null mutants is still unclear, but it could be secondary to the disruption of the ankle links in these mutants.

**Figure 5:**
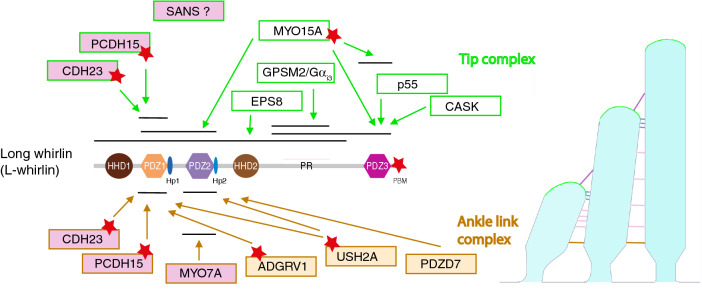
L-whirlin-interacting networks at the ankle-links and at stereocilia tips. Schematic diagram of the known interactions between L-whirlin and proteins of the ankle-link molecular complex (below) or proteins present at the tips of the tall stereocilia (above). The whirlin regions where these interactions take place are indicated by horizontal bars. The proteins implicated in Usher syndrome of type I and II are highlighted in pink and yellow, respectively. Red stars denote the presence of a C-terminal PDZ binding motif (PBM).

We also show that L-whirlin is present at the tips of the tall stereocilia of both IHCs and OHCs during hair bundle development and at mature stages. Inconsistencies in the L-whirlin immunolocalizations reported by different groups in mature hair cells might be explained by epitope masking for some antibodies. However, neither S-whirlin nor L-whirlin has so far been detected at the tips of the short or middle-sized stereocilia, which suggests that their presence at stereocilia tips is indeed restricted to the tall stereocilia. This also suggests that L-whirlin is not critical for the maturation of the mechanoelectrical transduction machinery, which is consistent with the mild auditory phenotype of L-whirlin-null mutant mice^46^. The control of F-actin polymerization at the stereocilia tips involves a molecular complex containing the epidermal growth factor receptor kinase substrate 8 (EPS8), espin, myosin III, and myosin XV, and presumably other proteins such as villin and fascin^28,46,47^. The presence of L-whirlin specifically at the tips of the tall stereocilia (as shown in this study) could contribute, together with the G protein subunit Gα_i3_ and the G-protein signaling modulator 2 (GPSM2), to confer this row its unique identity, which is in turn critical in establishing and maintaining differential row identity across the hair bundle^48^. GPSM2 interacts with the HHD2-PDZ3 linker region present in both whirlin isoforms^31^. In addition, it has been shown that S-whirlin functions as an adaptor between myosin XV and the two actin-regulatory proteins EPS8 and MAGUK protein p55^28,32,49^ (Fig. 5). Finally, the presence of both L-whirlin and the PCDH15 isoforms CD1 and CD2 at the tips of the tall stereocilia, and their physical interaction demonstrated here in vitro, raises the possibility that L-whirlin might be required to anchor a subpopulation of apical lateral links containing PCDH15 or CDH23 to the actin cores of these stereocilia. Indeed, the top-connector-associated proteins stereocilin, otogelin and otogelin-like have an abnormally extended distribution in the absence of PCDH15 or CDH23^17^. Accordingly, the impaired DPOAEs and frequency tuning in mutant mice lacking the L-whirlin isoform could reflect a loosening of the OHC hair bundle cohesiveness due to the loss of these apical lateral links, since the loss of OHC bundle cohesiveness abolishes DPOAEs, as occurs in stereocilin-null mutant mice which lack the top-connectors^16,17,50^.

In summary, our results support a role for L-whirlin as a submembrane binding partner of PCDH15, and possibly of CDH23, thereby extending the contributions of these cadherins to fibrous lateral links interconnecting the bases of the stereocilia during development, and the tips of the tallest stereocilia at developmental and mature stages.

## Materials and methods

### Antibodies

Rabbit polyclonal antibodies were produced against part of the specific C-terminal part of each of the PCDH15 isoforms (Anti-PCDH15-CD1: aa residues 1612–1726, Genbank accession no. Q99PJ1-1; anti-PCDH15-CD2: aa residues 1652–1790, Genbank accession no. Q99PJ1-10; anti-PCDH15-CD3: aa residues 1522–1682, Genbank accession no. Q99PJ1-18) and against either the N-terminal region specific to the L-whirlin isoform (L-whirlin NTR: aa residues 2–113, Genbank accession no. NP_082916) or against domains common to both S-whirlin and L-whirlin isoforms (pan-whirlin: aa residues 2–506; whirlin-PDZ3: aa residues 809–907, Genbank Accession no. NP_082916). The antigens coupled to an NHS column (GE Healthcare) were used for affinity purification of these antibodies from the immune serum.

The generation of antibodies in rabbit was accomplished by an external company (Agro-Bio, France).

### Western blot experiments

The cDNAs encoding the cytoplasmic regions of mouse CDH23, PCDH15-CD1, PCDH15-CD2, and PCDH15-CD3 (Genbank accession no. AY563163, Q99PJ1-1; Q99PJ1-10, and Q99PJ1-18, respectively), were cloned in modified pcDNA3 vectors with a Flag-tag-encoding sequence at their 5′ end. The cDNAs encoding the short and long isoforms of whirlin were cloned in PCMV vectors. The various recombinant proteins were produced in transfected HEK-293 cells. Cells were collected 48 h after transfection by centrifugation. The pellets were dissolved in Nupage Sample Buffer (Invitrogen) and submitted to 4–12% Nupage SDS-PAGE (Invitrogen). The separated proteins were blotted onto PVDF membranes (Millipore), and were processed for immuno-chemiluminescence detection (Pierce Biotechnology). An anti-flag monoclonal antibody (M2 Sigma-Aldrich, 1:500 dilution) was used to detect Flag-CDH23 cyto, Flag-PCDH15-CD1 cyto, Flag-PCDH15-CD2 cyto and Flag-PCDH15-CD3 cyto.

### Immunofluorescence and SEM experiments on the cochlear sensory epithelium

The affinity-purified antibodies were used for immunofluorescence experiments on whole-mount preparations of mouse organs of Corti. The images we present in our manuscript were all obtained from the mid-apical region of the cochlea (between 0.5 and 0.75 in a normalized cochlear axis pointing toward the apex). We used both ears from at least ten animals for each experiment. Cochlea were excised and the samples were fixed in phosphate-buffered saline (PBS) containing 4% paraformaldehyde for 1 h, then immersed in blocking buffer (20% normal goat serum (NGS) and 0.3% Triton X-100 in PBS) for 1 h at room temperature, and then incubated with the primary antibody in PBS containing 2% bovine serum albumin overnight in 4 °C. The fluorescent tagged secondary antibodies (Atto-488-conjugated goat anti-rabbit IgG fragment (1/200, Sigma-Aldrich) and Atto-565-conjugated phalloidin (0.8 μM, Sigma-Aldrich) for actin staining were applied and incubated for 1 h at room temperature. Samples were washed with PBS and covered with mounting medium (FluorSave Reagent; Calbiochem). Fluorescent immunolabelings were analyzed with a Zeiss LSM-700 confocal microscope.

For SEM immunolabelling, the cochlea was fixed for 1 h in a 2% PFA solution, after which a classical immunolabeling protocol was applied (by blockage for 1 h in PBS/20% NGS, 0,3% Triton X-100, RT, before exposition to primary antibodies at 4 °C, overnight). To detect the primary antibodies, we added protein A-conjugated 15 nm colloidal gold particles (EM Lab, Utrecht, The Netherlands; diluted 1/60 in PBS/BSA). Finally, samples were post-fixed for 1 h in 2.5% glutaraldehyde in PBS before proceeding to SEM.

### Harmonin and whirlin fragments

The cDNA sequences encoding the different fragments of harmonin and whirlin (harmonin-NTD-PDZ1: aa 1–192 residues, harmonin-PDZ2: aa residues 197–308, harmonin-PDZ3: aa residues 738–849, Genbank AF228925.1, full-length harmonin-a1, Genbank NM_023649, whirlin-HHD1: aa residues 30–121, whirlin-NTR: aa residues 2–147, whirlin-NTR-PDZ1: aa residues 2–265, whirlin-NTR-PDZ1-PDZ2: aa residues 2–368, whirlin-PDZ1: aa residues 137–228, whirlin-PDZ2: aa residues 272–368, whirlin-PDZ3: aa residues 821–915, Genbank Accession no Q80VW5) were amplified and cloned (Spe1-AscI) into a modified pGST//2 vector (derived from pGEX-4T1, Amersham) for expression in *E. coli* prokaryotic cells. Point mutations were introduced using QuickChange II CL Site-directed Mutagenesis Kit (Agilent Technologies). Proteins were produced in BL21 (DE3) codonPlus-RIPL *E. coli* cells. The gluthathione-S-transferase (GST)-tagged proteins were purified using Gluthathione Sepharose 4B (GE Healthcare). The GST-tag was removed using recombinant tobacco etch virus proteinase. All the proteins used in these studies have an extra 7aa (GAMGSTS) at their N-terminus. Proteins were further purified by size exclusion chromatography (Superdex 75 10/300 for most construct, Superdex 200 10/300 for Whirlin-PDZ3 and Sephacryl S-100 h 16/600 for whirlin-PDZ1 + Hp1).

### SPR experiments

Whirlin-PCDH15 SPR experiments were performed on a ProteOn XPR 36 system (Biorad) equilibrated with a buffer containing 250 mM NaCl, 50 mM Tris pH 7.5, 0.5 mM TCEP. N-terminal biotinylated peptides for the different ligands (PCDH15-CD1: biotin-TTDS-GIDVQPHSQSTSL; PCDH15-CD2: biotin- TTDS-GANSEQYNTAL; PCDH15-CD3: biotin-TTDS-MGPWNSLHIPMTKL; CDH23: biotin-TTDS-VIMESPLEITEL; TTDS: Trioxatridecan-succinamic acid as a spacer) were synthetized (Eurogentec – France) and captured by affinity on a Neutravidin- functionalized NLC sensor chip (Biorad), over which the different whirlin fragments were flowed as analytes at 20 μL/min for 2 min. For calculation of high to moderate affinity constants only the signals obtained using analytes at concentrations below 10 μM were included to exclude the possible impact of oligomerization of whirlin fragments. The steady-state SPR responses (*R*eq, measured experimentallly or determined by extrapolation of the association curves) were plotted against the concentration (*C*) of analyte and fitted using the following equation, *R*_eq_ = (*R*_max_ * *C*)/(*K*_*d*_ + *C*), where *K*_*d*_ is the equilibrium dissociation constant, and *R*_max_ the maximal binding capacity of the surface, using BIAevaluation 4.1 software (Biacore).

### Circular dichroism

All circular dichroism (CD) measurements were obtained using an Aviv 215 spectropolarimeter. Far-UV (195–260 nm) spectra were recorded at 20 °C on 20 μM whirlin samples in 250 mM KCl, 50 mM Tris pH 8, 1 mM TCEP-buffer, using a 0.2 mm path-length cylindrical cell. Ellipticity was measured every 0.5 nm with an averaging time of 2 s. Three successive spectrum scans of the protein sample were averaged and then the baseline spectrum of buffer is subtracted. We used CONTIN program for the far-UV CD spectrum quantitative decomposition^51^. The percentage of secondary structure content of each recombinant protein was calculated using the BeStSel online engine (available at https://bestsel.elte.hu/index.php).

### Animals

Animal experiments were carried out in accordance with European Community Council Directive 2010/63/UE under authorizations 2014–005 and dap170051 from the Institut Pasteur ethics committee for animal experimentation. A total of 254 cochleas from 127 mice were used in this study. Males and females were used indifferently.

*Pcdh15*^av3J/av3J^ mice*: Pcdh15*^av3J/av3J^ mice were obtained from Jackson Laboratories (Bar Arbor, ME).

*Pcdh15*^ex38-fl/ex38-fl^ mice: For *Pcdh15*^ex38-fl/ex38-fl^ mice see^40^.

### *Pcdh15*^ex35-fl/ex35-fl^ mice and *Pcdh15*^ex39-fl/ex39-fl^ mice

Mutant mice were generated by inserting LoxP sites upstream and downstream from *Pcdh15* exon 35 or exon 39, respectively. A Neomycin-resistance (neo) cassette flanked with Frt sites as selectable marker was introduced downstream of the concerned exon as selectable marker (See Fig. S3). Embryonic stem cells (ES cells) from 129S1/SvlmJ mice were electroporated to introduce the targeting construct. Based on their resistance to G418, positive ES cells were selected and injected into blastocysts from C57BL/6J mice to obtain chimeric mice. After germline transmission, by crossing with C57BL/6J mice producing Flp recombinase the neo cassette was removed in the offspring. These mice *Pcdh15*^ex35-fl/ex35-fl^ mice (MGI:6389238) and *Pcdh15*^ex39-fl/ex39-fl^ mice (MGI:6389239) only carry LoxP sites flanking the concerned exon and behave similarly to wild-type mice (*Pcdh15*^+/+^). To delete the concerned exon in all cells these mice were crossed with *PGK-cre* transgenic mice carrying the *cre* recombinase gene under control of then early and ubiquitously active phosphoglycerate kinase-1 gene promoter^52^. For genotyping of these recombinant mice two PCR amplifications experiments were carried out, using the oligonucleotides used as primers and the amplicon sizes are listed below (Table 2). Mixed C57BL/6–129/Sv genetic backgrounds were used for this study.

**Table 2:** Oligonucleotides used as PCR primers for genotyping the different mutant mice recombinant for one of the *Pcdh15* exons.

Mutant mouse model	Oligo	Sequence	Amplicon size
*Pcdh15*^ex35-fl/ex35-fl^	Ef1-5702	5′-gtcgtaaccctcaaagcggagcac-3′	WT: 291 bp
	Er1-5704	5′-ccatcaacaaggtcaacacgcctttg-3′	Floxed: 397 bp
	Lf2-5700	5′-gttctgggcaccctccagaaagatg-3′	WT: 2131 bp
	Er1-5702	5′- ccatcaacaaggtcaacacgcctttg-3′	Del: 324 bp
*Pcdh15*^ex39-fl/ex39-fl^	EF-5412	5′-ggttggatcttgaattagtcttttcc-3′	WT: 297 bp
	Er-5414	5′-cattgtggatactatgtaacttcagg-3′	Floxed: 399 bp
	Lf-5708	5′-gctttctgctgaacttgctgaatgg-3′	WT: 1383 bp
	Er-5413	5′-cacctggactgctttgtaagc-3′	Del: 263 bp

## Supplementary information

Supplementary information
