# Supplementary material for: Interaction of protocadherin-15 with the scaffold protein whirlin supports its anchoring of hair-bundle lateral links in cochlear hair cells
Source: Sci Rep. 2020 Oct 2;10:16430. doi: 10.1038/s41598-020-73158-1 (PMC7532178; doi:10.1038/s41598-020-73158-1)
Supplement: Supplementary file 1 — Supplementary information [file 41598_2020_73158_MOESM1_ESM.pdf]

Interaction of the scaffolding protein whirlin with protocadherin-15 supports its anchoring of hair-bundle lateral links in cochlear hair cells

Vincent Michel[1,2,3,4‡], Elise Pepermans[1,2,3,5,‡], Jacques Boutet de Monvel[1,2,3,4], Patrick England[6], Sylvie Nouaille[1,2,3,4], Alain Aghaie[1,2,3,4,7], Florent Delhommel[3,8,9], Nicolas Wolff[3,8], Isabelle Perfettini[1,2,3], Jean-Pierre Hardelin[1,2,3], Christine Petit[1,2,3,4,5,7,10,*], Amel Bahloul[1,2,3,11,*]

[1]Unité de génétique et physiologie de l'audition, Institut Pasteur, 75015 Paris, France

[2]Institut National de la Santé et de la Recherche Médicale, UMRS 1120, 75015 Paris, France

[3]Complexité du vivant, Sorbonne Université, 75005 Paris, France

[4]Institut de l'audition, Centre de l'Institut Pasteur, 75012 Paris, France

[5]Current address: Center for proteomics, University of Antwerp, 2020 Antwerp, Belgium

[6]Plateforme de biophysique moléculaire, Institut Pasteur, Paris, France.

[7]Syndrome de Usher et autres atteintes rétino-cochléaires, Institut de la vision, 75012 Paris, France.

[8]Unité Récepteurs-Canaux, Institut Pasteur, 75015 Paris, France

[9]Current address: Institute of structural biology, Helmholtz Zentrum München, Germany

[10]Collège de France, 75005 Paris, France

[11]Current address: Department of otolaryngology - Head and neck surgery, Stanford university, Stanford, California, USA.

†These authors contributed equally to this work

*Corresponding authors. E-mail: amel.bahloul@stanford.edu and christine.petit@pasteur.fr

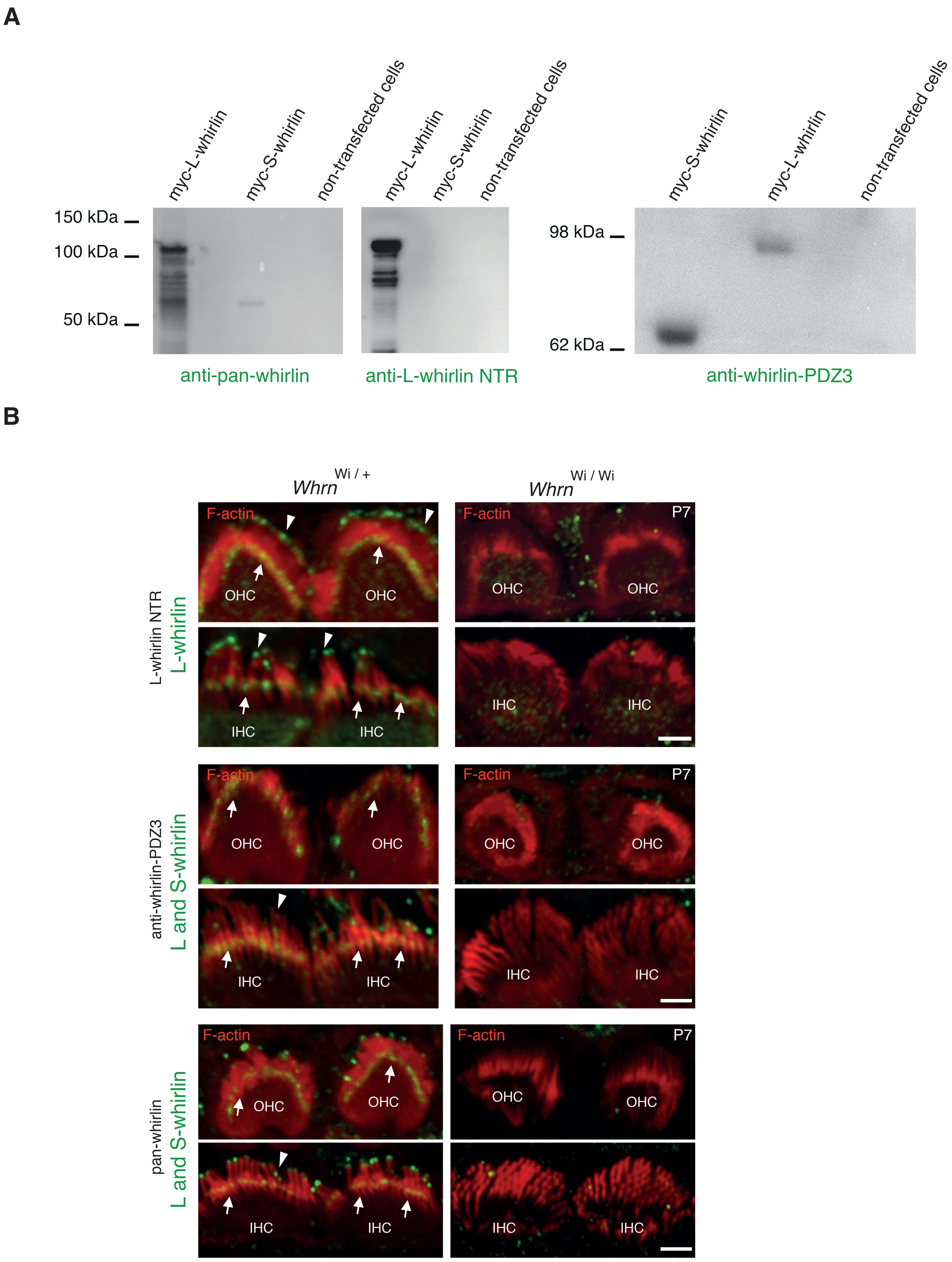

SUPPLEMENTARY FIGURE 1

A

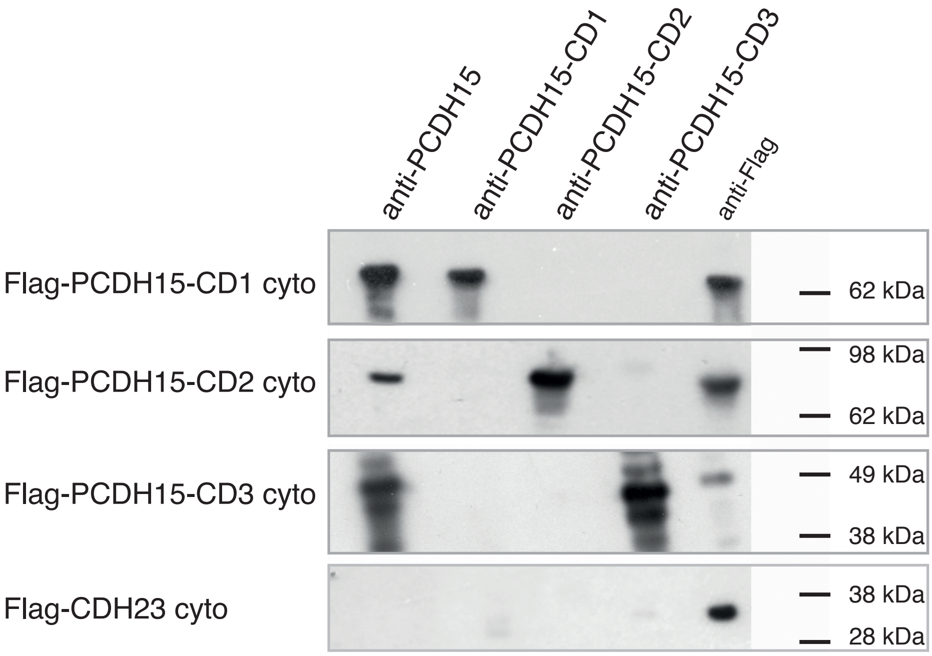

B

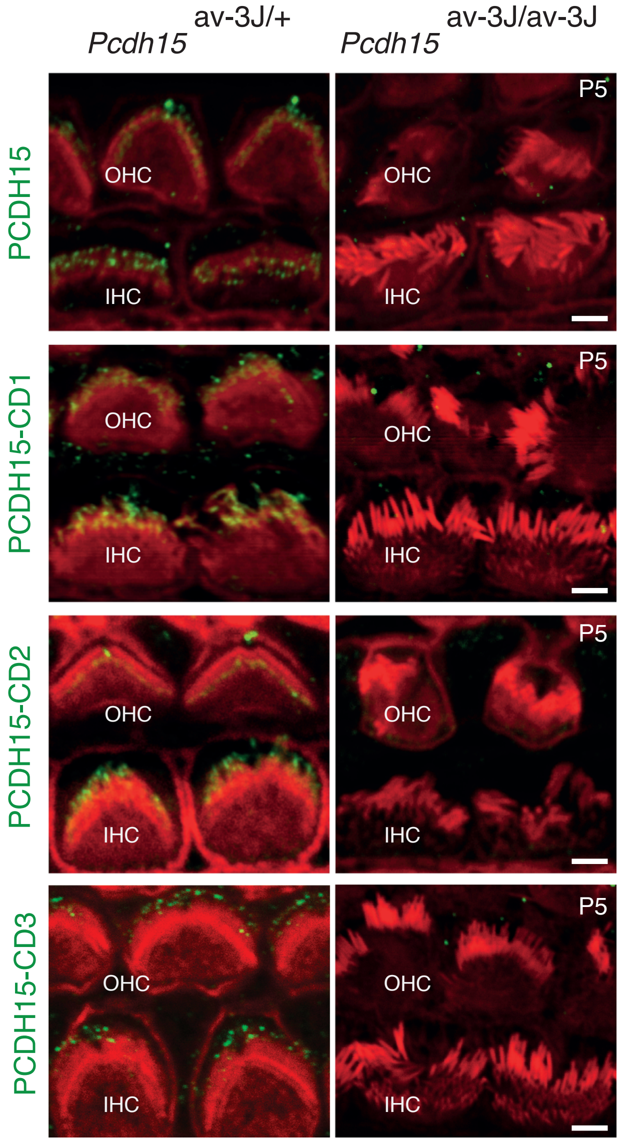

SUPPLEMENTARY FIGURE 2

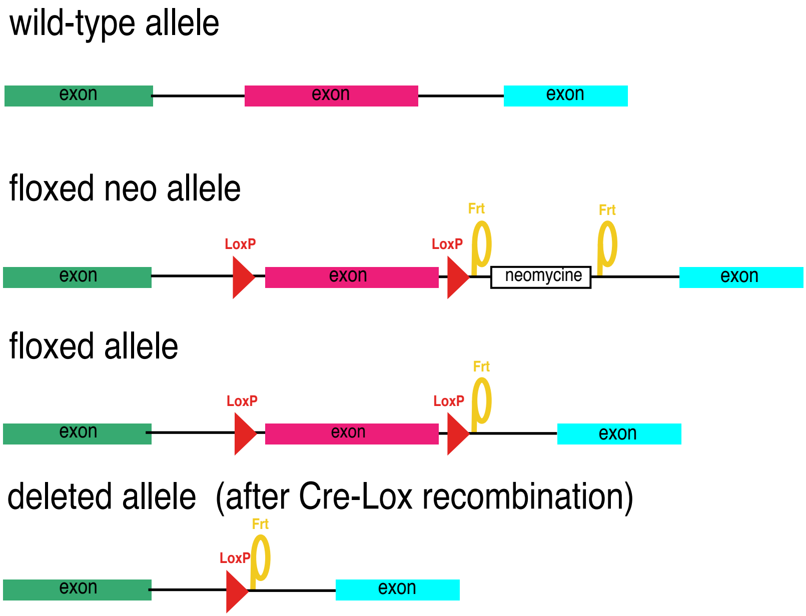

SUPPLEMENTARY FIGURE 3

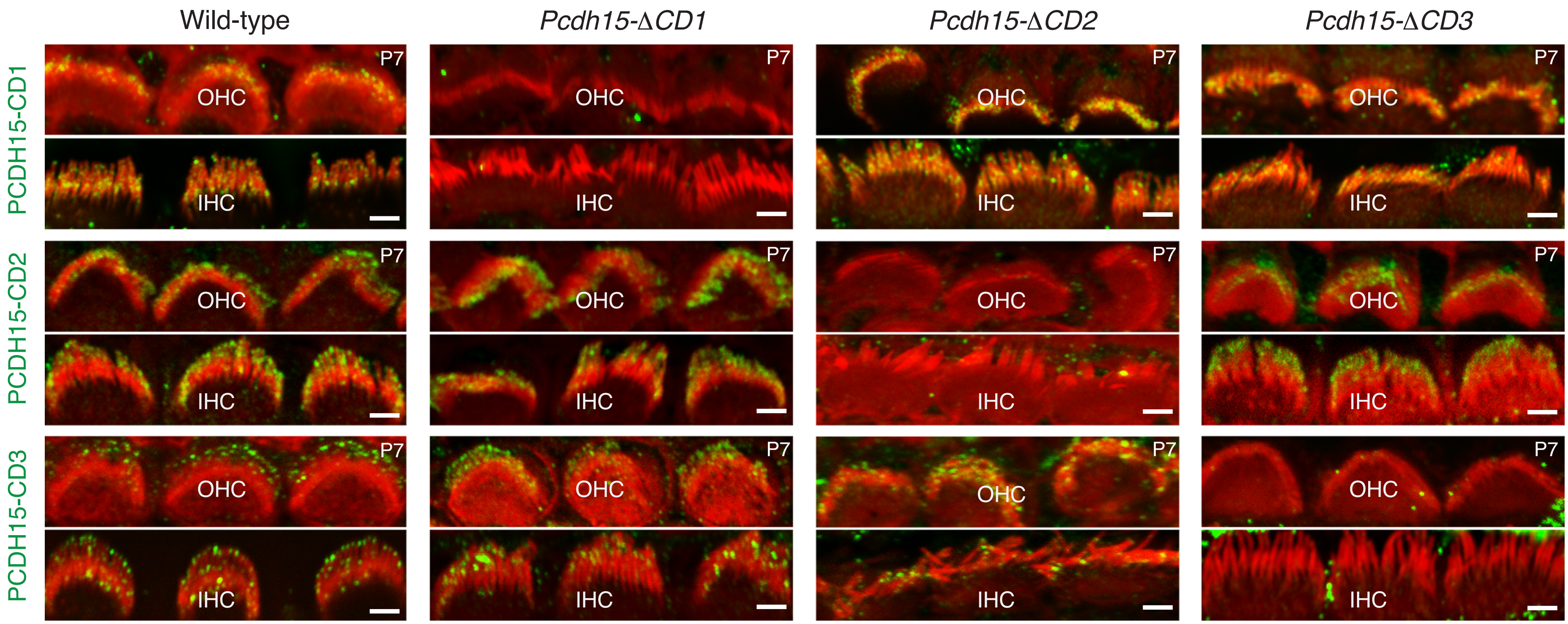

SUPPLEMENTARY FIGURE 4

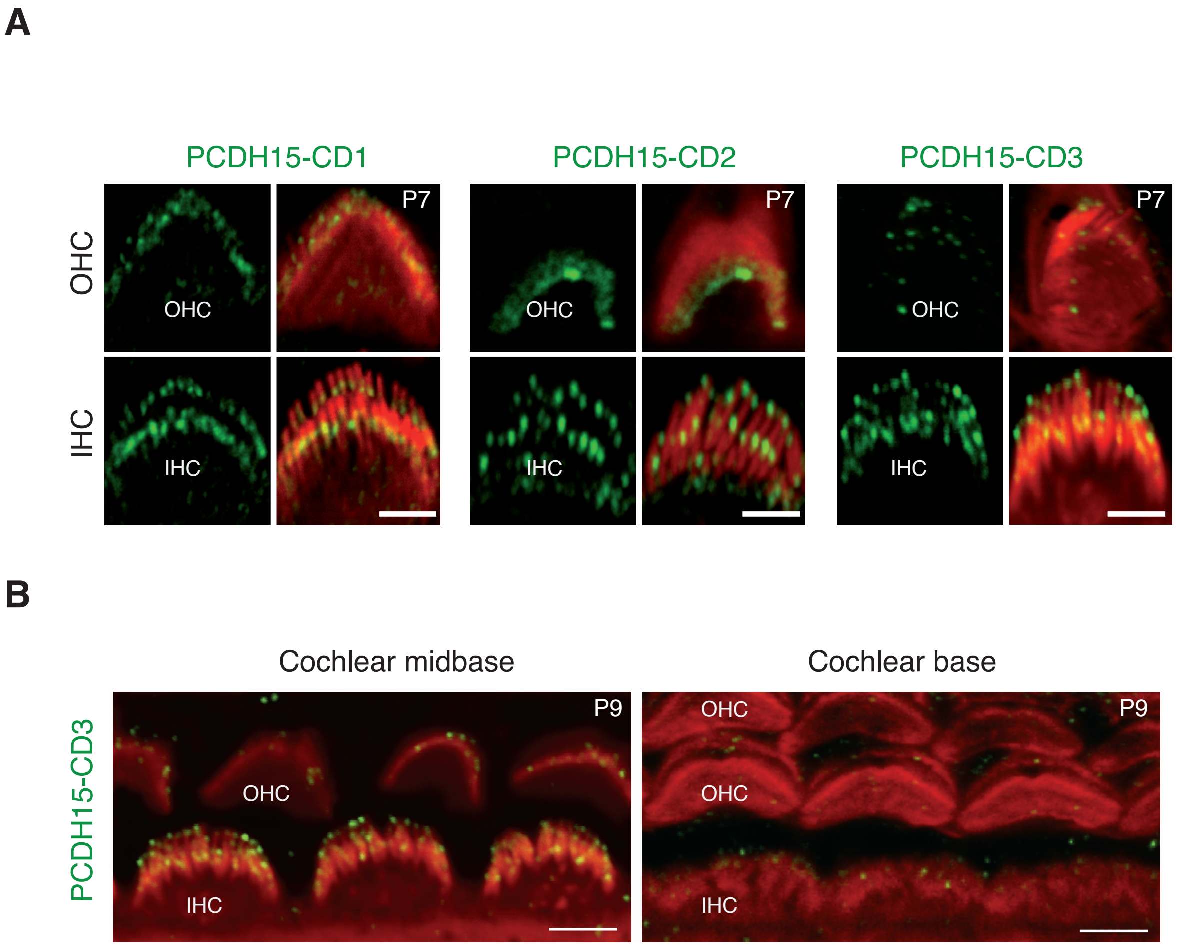

SUPPLEMENTARY FIGURE 5

A

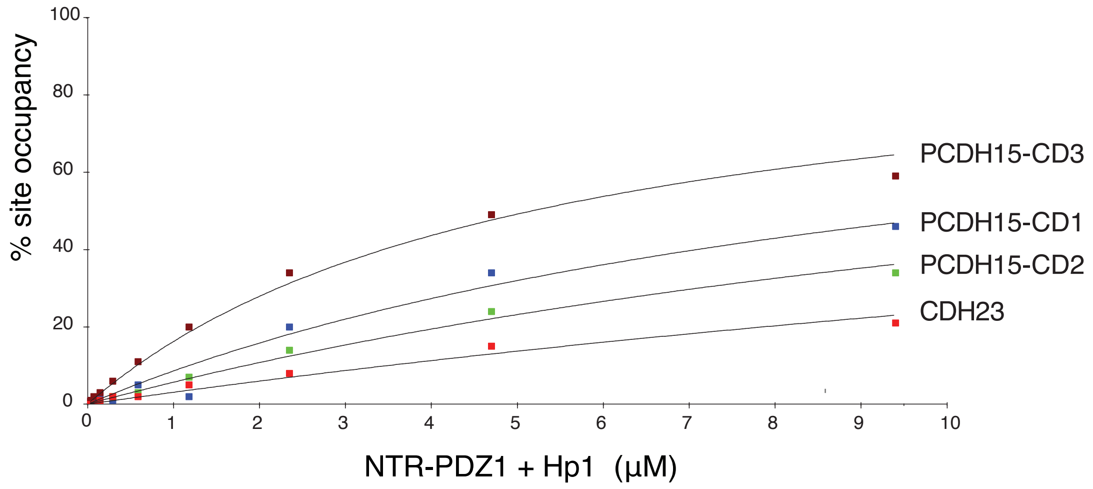

B

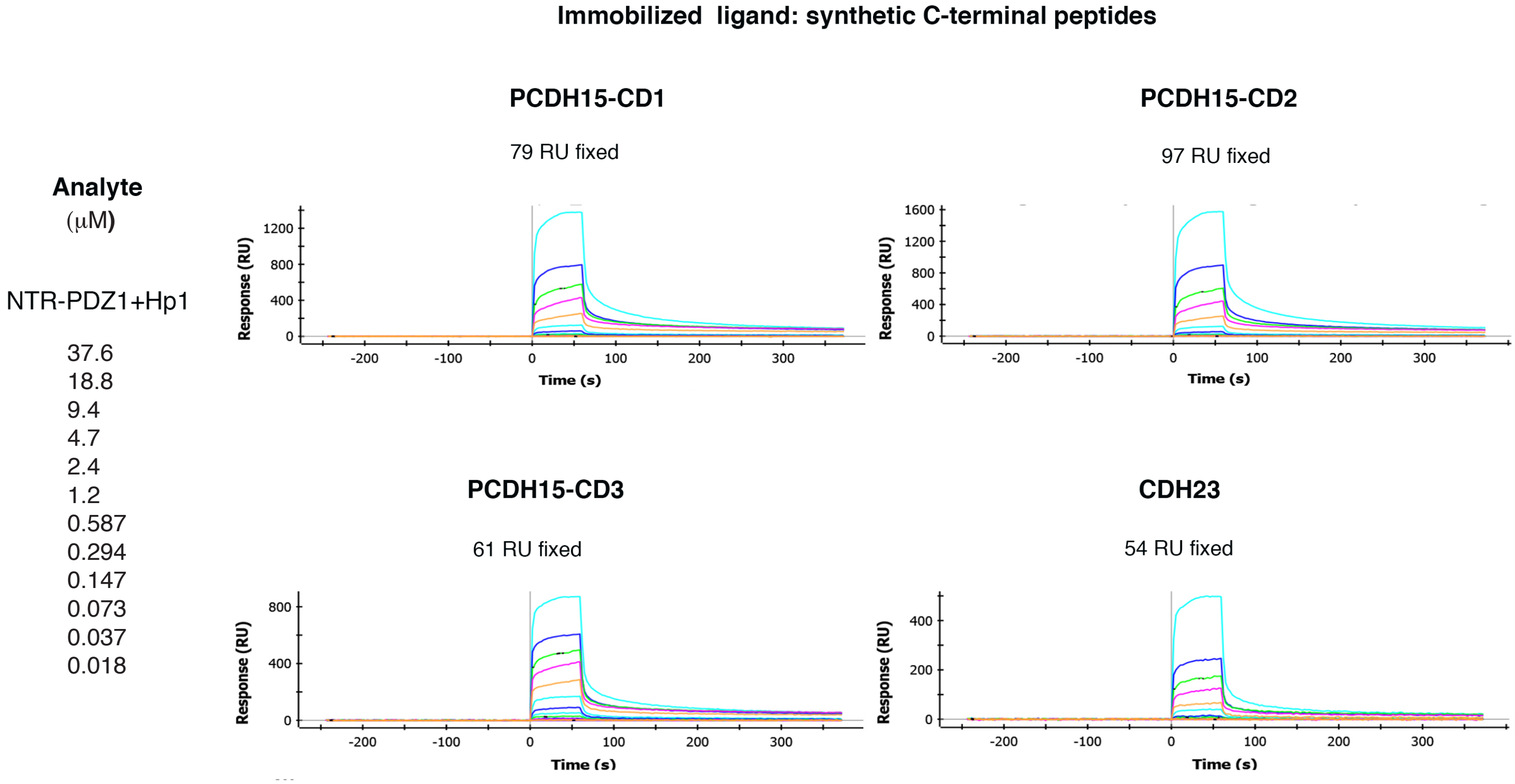

SUPPLEMENTARY FIGURE 6

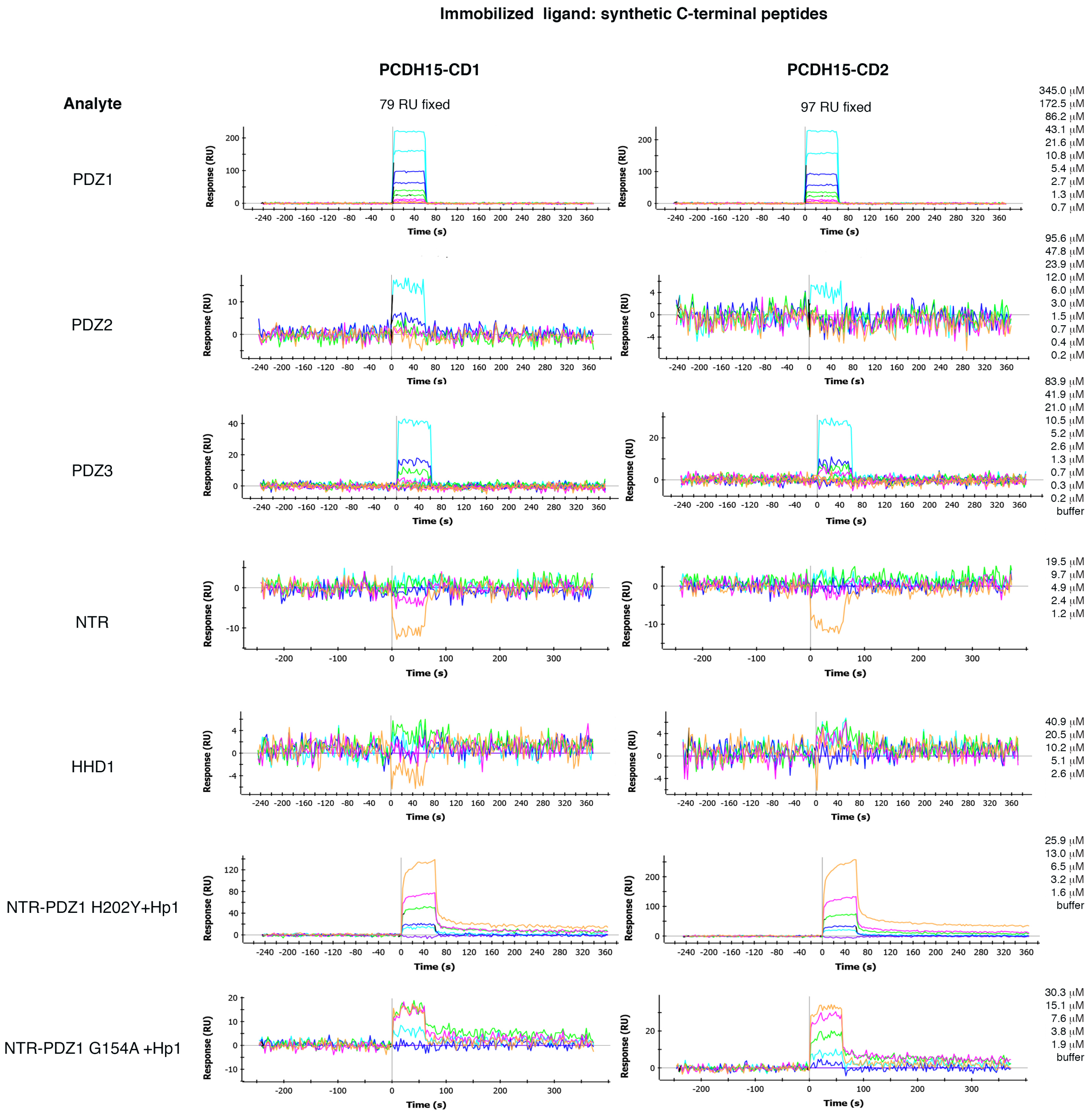

SUPPLEMENTARY FIGURE 7

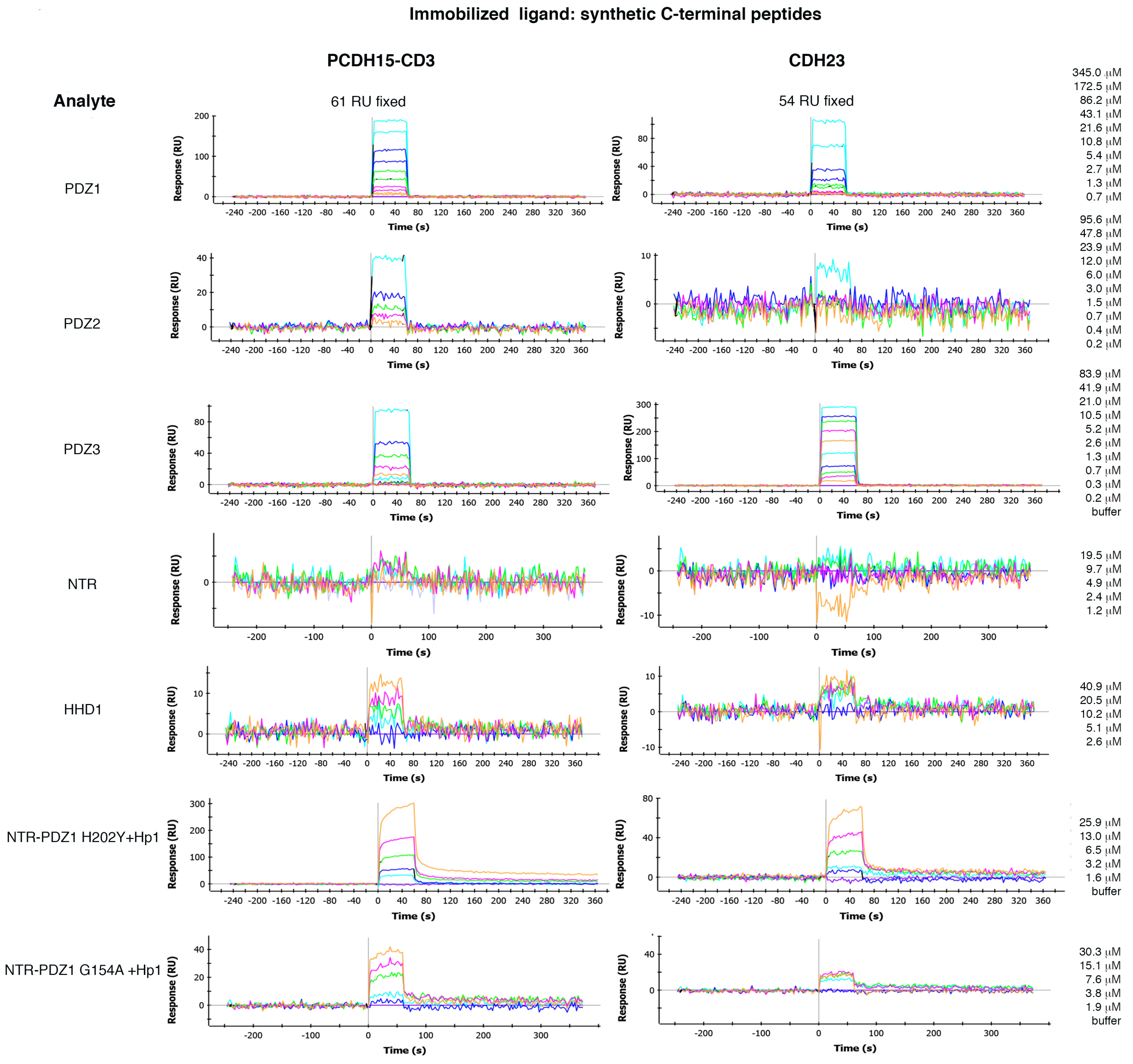

SUPPLEMENTARY FIGURE 8

A

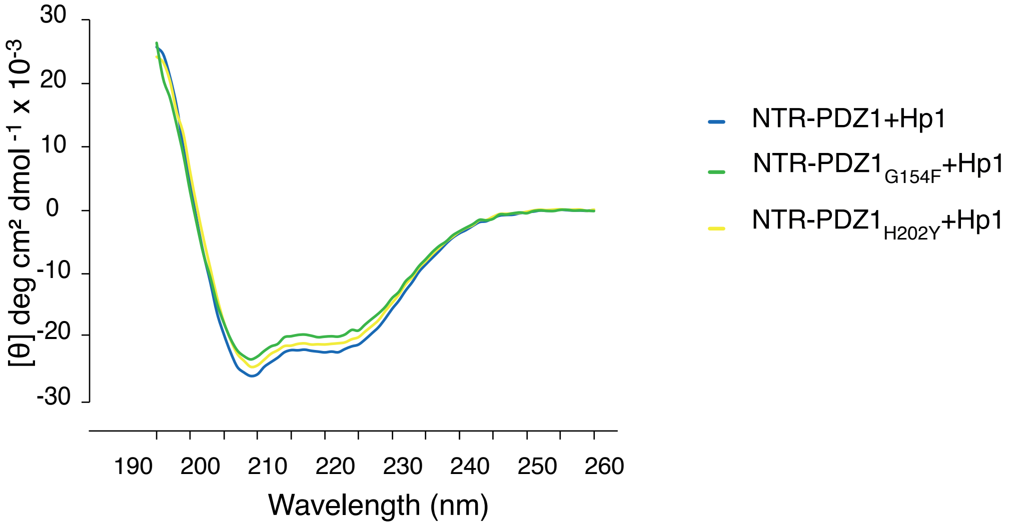

B

	NTR-PDZ1+Hp1	NTR-PDZ1$_{H202Y}$+Hp1	NTR-PDZ1$_{G154F}$+Hp1
Helix (%)	56.4	57.3	52.3
Antiparallel β-sheet (%)	2.8	2.3	3.1
Turn (%)	9.1	9.4	10.2
Others (%)	31.7	31.0	34.3

SUPPLEMENTARY FIGURE 9

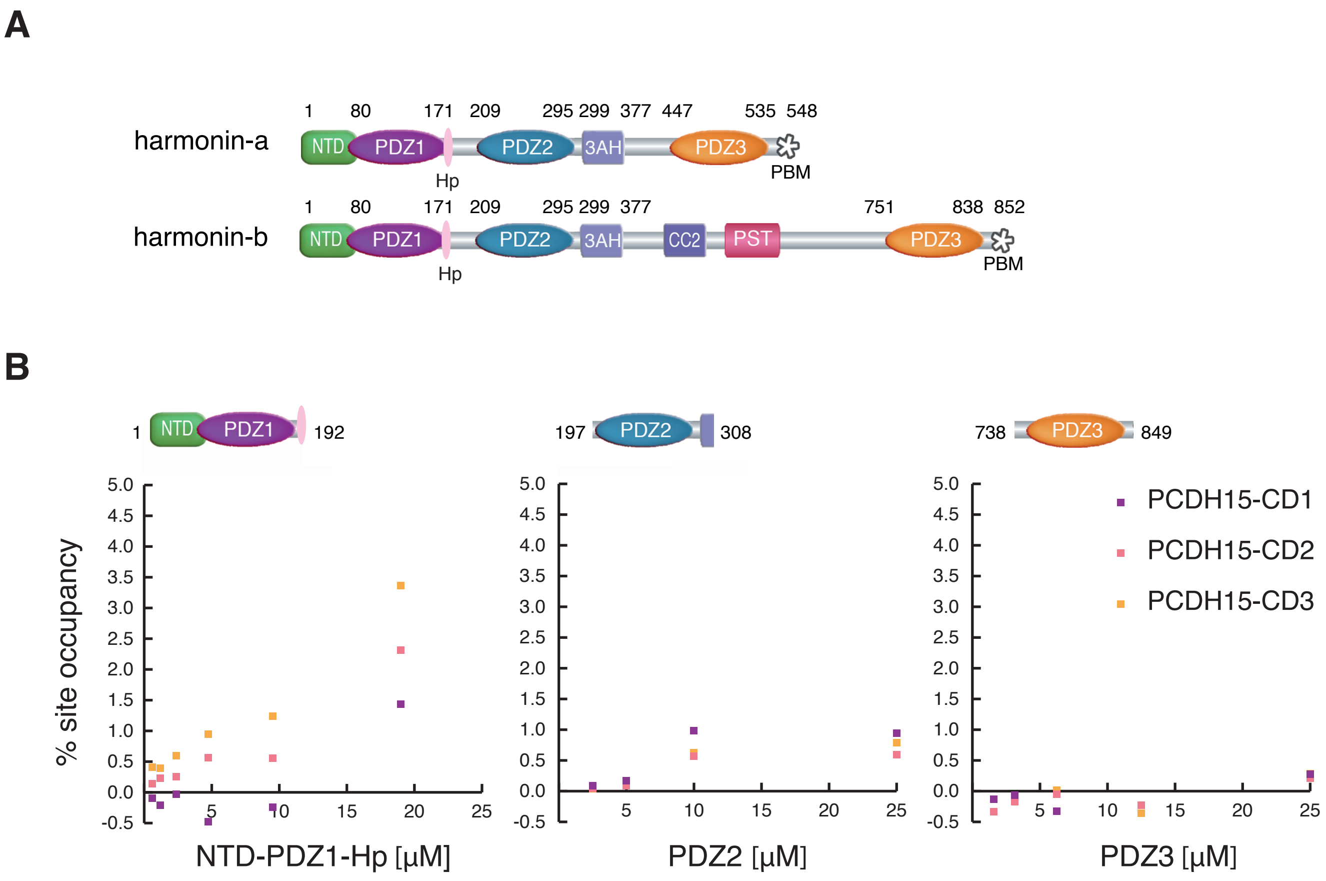

SUPPLEMENTARY FIGURE 10

TABLE S1: List of anti-whirlin antibodies previously used for immunofluorescence experiments in mice

Antigen	Isoforms	Name	Localization in hair bundle	Stage	Reference
**H61-L920 End**	L and S-whirlin	CIP98			1
**V735-R750**	L and S-whirlin	WCt1	Cochlear and vestibular HC		2
**H61-L920 End**	L and S-whirlin	CIP98	Tip and base in IHC and OHC	Developmental (< P10)	3
**R362-P509**	L and S-whirlin	HL5136	Tip in IHC and OHC	Developmental (P7)	4
**H722-T825**	L and S-whirlin	HL5141	Tip in IHC	Mature	4
**F732-A811**	L and S-whirlin	WHRN_C	Tip and base IHC and OHC	Developmental (P4)	5
**F732-A811**	L and S-whirlin	WHRN_C	Tip in IHC	Mature	5
**A796-N815**	L and S-whirlin	PB584	Tip in IHC midway of middle stereocilia of IHC and OHC	Developmental (P10)	6
**M1-G320 / E315-I580 NP_082916**	L-whirlin / L and S-whirlin	PDZ320 / PDZIE	Base in IHC and OHC		7
**M1-Y234 N718-L907 End NP_056219.3**	L-whirlin / L and S-whirlin	GP66 GP68	Tip and base in IHC and OHC		8
**M1-G320 / E315-I580 NP_082916**	L-whirlin / L and S-whirlin	PDZ320 / PDZIE	Tip and base in IHC and OHC		9
**T48-C62 + H255-T269**	L-whirlin	WNt1A and WNt1B	Cochlear and vestibular HC		2
**M1-R124**	L-whirlin	WHRN_N	Tip and base in IHC Base in OHC	Developmental (P4)	5
**M1-R124**	L-whirlin	WHRN_N	Tip in IHC	Mature	5
**S248-D265**	L-whirlin	PB595	midway of middle row stereocilia of IHC and OHC	Developmental (P10)	6

REFERENCES

1. Yap, C.C., *et al.* CIP98, a novel PDZ domain protein, is expressed in the central nervous system and interacts with calmodulin-dependent serine kinase. *J Neurochem* **85**, 123-134 (2003).
2. Mburu, P., *et al.* Defects in whirlin, a PDZ domain molecule involved in stereocilia elongation, cause deafness in the whirler mouse and families with DFNB31. *Nature genetics* **34**, 421-428 (2003).
3. Delprat, B., *et al.* Myosin XVa and whirlin, two deafness gene products required for hair bundle growth, are located at the stereocilia tips and interact directly. *Human molecular genetics* **14**, 401-410 (2005).
4. Belyantseva, I.A., *et al.* Myosin-XVa is required for tip localization of whirlin and differential elongation of hair-cell stereocilia. *Nat Cell Biol* **7**, 148-156 (2005).
5. Mathur, P.D., *et al.* A study of whirlin isoforms in the mouse vestibular system suggests potential vestibular dysfunction in DFNB31-deficient patients. *Hum Mol Genet* **24**, 7017-7030 (2015).
6. Ebrahim, S., *et al.* Alternative Splice Forms Influence Functions of Whirlin in Mechanosensory Hair Cell Stereocilia. *Cell Rep* **15**, 935-943 (2016).
7. Yang, J., *et al.* Ablation of whirlin long isoform disrupts the USH2 protein complex and causes vision and hearing loss. *PLoS genetics* **6**, e1000955 (2010).
8. Grati, M., *et al.* Localization of PDZD7 to the stereocilia ankle-link associates this scaffolding protein with the Usher syndrome protein network. *J Neurosci* **32**, 14288-14293 (2012).
9. Zou, J., *et al.* Deletion of PDZD7 disrupts the Usher syndrome type 2 protein complex in cochlear hair cells and causes hearing loss in mice. *Human molecular genetics* **23**, 2374-2390 (2014).

FIGURE LEGENDS

Figure S1. Specificity of the anti-whirlin antibodies.

A. Western blot analysis showing the specificity of the anti-L-whirlin NTR antibody (left), of the anti-pan-whirlin antibody (middle), and of the anti-whirlin PDZ3 (right) on lysates from transfected HEK293 cells producing myc-tagged L-whirlin (myc-L-whirlin) or S-whirlin (myc-S-whirlin), and from untransfected HEK cells used as negative control.

B. Confocal microscopy images of OHC and IHC hair bundles from P7 wild-type and *Whrn*$^{wi/wi}$ mice immunostained for either L-whirlin (anti-L-whirlin-NTR) or both L-and S-whirlin isoforms (anti-whirlin-PDZ3 and anti-pan-whirlin) (green) and stained for actin (red). With the three antibodies, the whirlin immunoreactivity is detected at the base (white arrows) of stereocilia and at the tips of OHC and IHC tall stereocilia (arrowheads) in the wild-type mouse, but not in the *Whrn*$^{wi/wi}$ mouse. Unspecific labelling surrounding the hair cells and at the cuticular plate (top right panel) is observed. Scale bar: 2 µm.

Figure S2. Specificity of the anti-PCDH15 antibodies.

A. Western blot showing the specificity of the anti-PCDH15-CD1, anti-PCDH15-CD2, and anti-PCDH15-CD3 antibodies on lysates from transfected HEK293 cells producing the flag-tagged cytoplasmic domains of PCDH15-CD1 (Flag-PCDH15-CD1cyto), PCDH15-CD2 (Flag-PCDH15-CD2cyto), PCDH15-CD3 (Flag-PCDH15-CD3cyto), or CDH23 (Flag-CDH23cyto).

B. Confocal microscopy images of OHC and IHC hair bundles. Left panel: P5 wild-type and *Pcdh15*$^{av\text{-}3J/av\text{-}3J}$ mice immunostained for PCDH15-CD1, PCDH15-CD2 and PCDH15-CD3 respectively (green) and stained for actin (red). The immunostainings are detected in stereocilia of the wild-type mouse, but not of the *Pcdh15*$^{av3J/av3J}$ mouse. Scale bar: 2 µm.

Figure S3: **Generation of knockout mice specifically lacking PCDH15-CD1 or PCDH15-CD3.**

Generation of knockout mice lacking Pcdh15 exon 35 (PCDH15Δ CD1) or exon 39 (PCDH15Δ CD3); Schematic of the exon-35 and exon-39 -recombinant alleles of Pcdh15 (see Methods).

Figure S4. Specificity of the anti-PCDH15 antibodies.

Confocal microscopy images of P7 OHC and IHC hair bundles of P7 wild-type mice, PCDH15-Δ CD1 (specifically lacking PCDH15-CD1), PCDH15-Δ CD2 (specifically lacking PCDH15-CD2) and PCDH15-Δ CD3 (specifically lacking PCDH15-CD3) mice, immunostained for PCDH15-CD1, PCDH15-CD2, and PCDH15-CD3, respectively (green), and stained for actin (red). PCDH15-CD1 staining is observed in the hair bundles of wild-type, PCDH15-Δ CD2, and PCDH15-Δ CD3 mice, but not of PCDH15-Δ CD1 mice. PCDH15-CD2 staining is observed in the hair bundles of wild-type, PCDH15-Δ CD1, and PCDH15-Δ CD3 mice, but not of PCDH15-Δ CD2 mice. PCDH15-CD3 staining is observed in the hair bundles of wild-type, PCDH15-Δ CD1, and PCDH15-Δ CD2 mice, but not of PCDH15-Δ CD3 mice. Scale bar: 2 μm.

Figure S5. Distribution of the different PCDH15 isoforms in immature hair cells of the mouse cochlea.

A. Confocal microscopy images of OHC and IHC hair bundles (OHC above, IHC below) immunostained for PCDH15-CD1, PCDH15-CD2, or PCDH15-CD3 (green), and stained for actin (red) on P7. In OHCs, the CD1, CD2, and CD3 immunolabelings are located at the apices of all stereocilia. In IHCs, the CD2 and CD3 immunolabelings are located at the apices of the middle-sized and tall stereocilia, whereas the CD1 immunolabeling is restricted to a subapical region of the tall stereocilia.

B. Confocal microscopy images of OHC and IHC hair bundles immunostained for PCDH15-CD3 (green) and stained for actin (red) on P9. The PCDH15-CD3 immunolabeling has almost completely disappeared at the cochlear base, but is still detected in a more apical region of the cochlea, in agreement with the baso-apical gradient of cochlear maturation.

Figure S6. Fitting of steady-state SPR response (steady-state signal vs analyte concentration) and concentration dependence curves (signal vs time) for SPR experiments using NTR-PDZ1-Hp1 as analyte.

A. Global fit of the interaction SPR-sensorgrams. The steady-state SPR responses (Req, experimental or extrapolated) were plotted against the concentration (C) of analyte and fitted using

the following equation, $R_{eq} = (R_{max} * C)/(K_d + C)$, where K_d is the equilibrium dissociation constant, and R_{max} the maximal binding capacity for the specific analyte.

B. SPR-sensorgrams showing the SPR response to different concentrations of Nter-PDZ1-Hp1 as an analyte on the different immobilized peptides as ligands

Different concentrations (left panel) of whirlin construct (analyte) were injected over 79 RU (response unit), 97 RU, 61 RU, and 54 RU of immobilized peptides (ligand) for PCDH15-CD1, PCDH15-CD2, PCDH15-CD3, and CDH23 respectively.

Figure S7. Concentration dependence curves (signal vs time) for SPR experiments using different whirlin constructs as analytes on immobilized PCDH15-CD1 and PCDH15-CD2 peptides as ligands.

Different concentrations (right panel) of each whirlin construct (analyte) were injected over 79 RU (response unit) and 97 RU, of immobilized peptides (ligands) for PCDH15-CD1 and PCDH15-CD2 respectively. The SPR-sensorgrams show the SPR response to different concentrations of the whirlin constructs as analytes on the different immobilized peptides as ligands.

Figure S8. Concentration dependence curves (signal vs time) for SPR experiments using different whirlin constructions as analytes on immobilized PCDH15-CD3 and CDH23 peptides as ligands

Different concentrations (right panel) of each whirlin construct (analyte) were injected over 61 RU (response unit) and 54 RU, of immobilized peptides (ligands) for PCDH15-CD3 and CDH23, respectively. The SPR-sensorgrams show the SPR response to different concentrations of the whirlin constructions as analytes on the different immobilized peptides as ligands.

Figure S9. Circular dichroism spectra of wild-type and mutated NTR-PDZ1-Hp1 whirlin fragment.

A. The overall secondary structure of NTR-PDZ1 + Hp1 whirlin fragment is not altered by the G154F and H202Y amino acid substitutions in PDZ1.

B. Percentage of secondary structure content of each recombinant protein calculated using the BeStSel online engine (http://bestsel.elte.hu/index.php).

Figure S10. Interaction between the PCDH15 C-terminal peptides and harmonin.

A. Diagram of harmonin isoforms. Abbreviations: NTD: N-terminal domain; PDZ: postsynaptic density, disc large, zonula occludens; 3AH: three alpha-helices; CC: coiled-coil; PST: proline–serine–threonine rich; PBM: PDZ domain-binding motif; Hp: hairpin extension of the PDZ1 domain.

B. Affinity titration by surface plasmon resonance showing RU responses (% site occupancy) to different concentrations of NTD-PDZ1, PDZ2, and PDZ3 fragments of harmonin, on the C-terminal peptides of the three PCDH15 isoforms (CD1, CD2, and CD3). Very low affinity interactions were found with NTD-PDZ1-Hp (the supramodule formed by the N-terminal globular domain of harmonin, PDZ1, and its hairpin extension), and with PDZ2 (K_d > 200 μM), and no interaction could be detected with PDZ3 (K_d > 1 mM).

Table S1. List of the anti-whirlin antibodies reported in the literature

The whirlin sequence used for amino-acid numbering of the antigenic fragments is Genbank accession number Q80VW5, except if otherwise specified.
